# Supplementary material for: Genetic optimisation of bacteria-induced calcite precipitation in Bacillus subtilis
Source: Microb Cell Fact. 2021 Nov 18;20:214. doi: 10.1186/s12934-021-01704-1 (PMC8600894; doi:10.1186/s12934-021-01704-1)
Supplement: Supplementary file 1 — Additional file 1. Heterologous expression of Sporosarcina pasteurii urease in Bacillus subtilis. Urease activity of strains of B. subtilis carrying different complements of urease genes, determined by qualitative urease assay in colourimetric test broth. [file 12934_2021_1704_MOESM1_ESM.pdf]

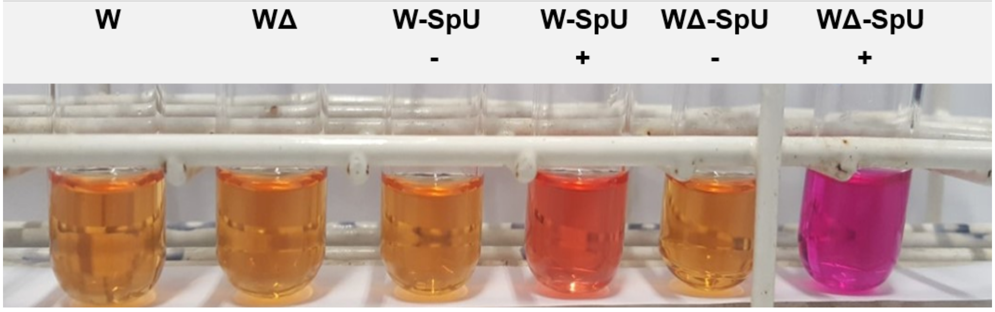

Additional file 1. Heterologous expression of *Sporosarcina pasteurii* urease in *Bacillus subtilis.* Strains of *B. subtilis* W168 (W) or a derived *ureABC-deletion* strain (WΔ) were transformed with a xylose-inducible plasmid containing the *S. pasteurii* urease gene cluster (SpU). Rapid urease test broth with or without addition of 0.2% xylose (+, -) to induce urease gene expression were inoculated with cells taken from growth on solid media and incubated at 37°C for 2 days. Urease activity is observed as a yellow-to-pink colour change of the medium resulting from a pH increase due to the release of ammonia from urea.
