# Supplementary material for: Genetic optimisation of bacteria-induced calcite precipitation in Bacillus subtilis
Source: Microb Cell Fact. 2021 Nov 18;20:214. doi: 10.1186/s12934-021-01704-1 (PMC8600894; doi:10.1186/s12934-021-01704-1)
Supplement: Supplementary file 2 — Additional file 2. Urease activity on solid media following heterologous expression of Bacillus paralicheniformis urease genes in B. subtilis. Urease activity of strains of B. subtilis carrying different complements of urease genes, determined by qualitative urease assay on agar plates containing the pH indicator phenol red. [file 12934_2021_1704_MOESM2_ESM.pdf]

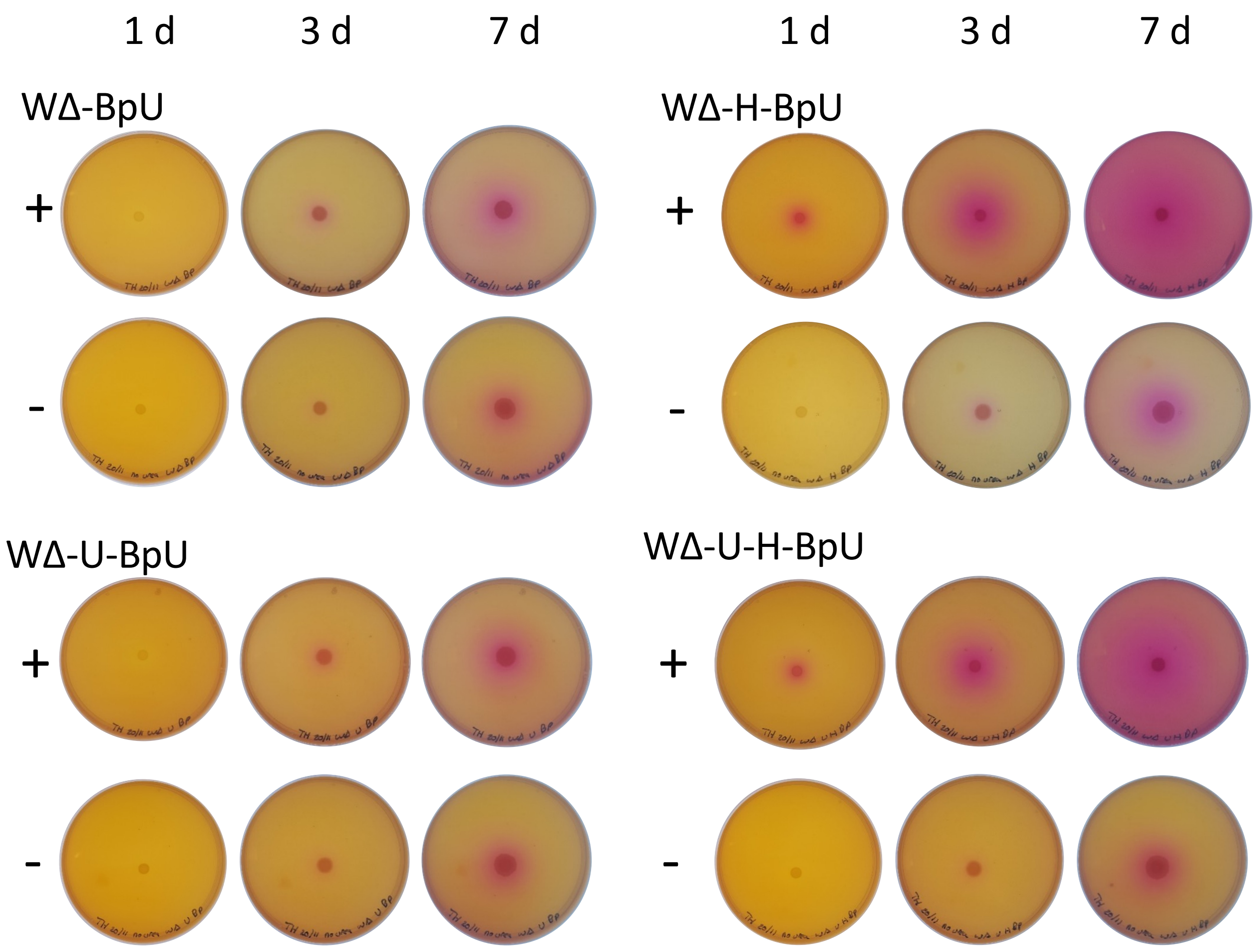

Additional file 2. Urease activity on solid media and precipitation assay following heterologous expression of *Bacillus paralicheniformis* urease genes in *B. subtilis.* Cells of a *ureABC* deletion strain of *B. subtilis W168* (WΔ) containing expression constructs for *B. paralicheniformis ureABCEFGD* (BpU), *ureH* (H), UT (U) or combinations thereof were spotted onto LBC agar plates with (+) or without (-) urea. The plates contained phenol red and had been adjusted to an initial pH of 7.2. Plates were incubated at 30°C and photographed after 1, 3 and 7 days as indicated. Urease activity is visible as a stronger development of pink colouration as compared to the urea-free control; the slight pink colouration immediately surrounding the bacterial growth is due to general metabolic activity. Results shown are representative of three independent experiments.
