# Supplementary material for: Genetic optimisation of bacteria-induced calcite precipitation in Bacillus subtilis
Source: Microb Cell Fact. 2021 Nov 18;20:214. doi: 10.1186/s12934-021-01704-1 (PMC8600894; doi:10.1186/s12934-021-01704-1)
Supplement: Supplementary file 3 — Additional file 3. Engineered urease and biomineralisation activity in B. subtilis NCIB3610. (A) urease activity of strains derived from B. subtilis NCIB3610 carrying different complements of urease genes, determined by qualitative urease assay on agar plates containing the pH indicator phenol red. (B-D) Biomineralisation of the two strains from panel A with the highest urease activity, comparing precipitation on LBC, B4 and biofilm-promoting LBGMC media. [file 12934_2021_1704_MOESM3_ESM.pdf]

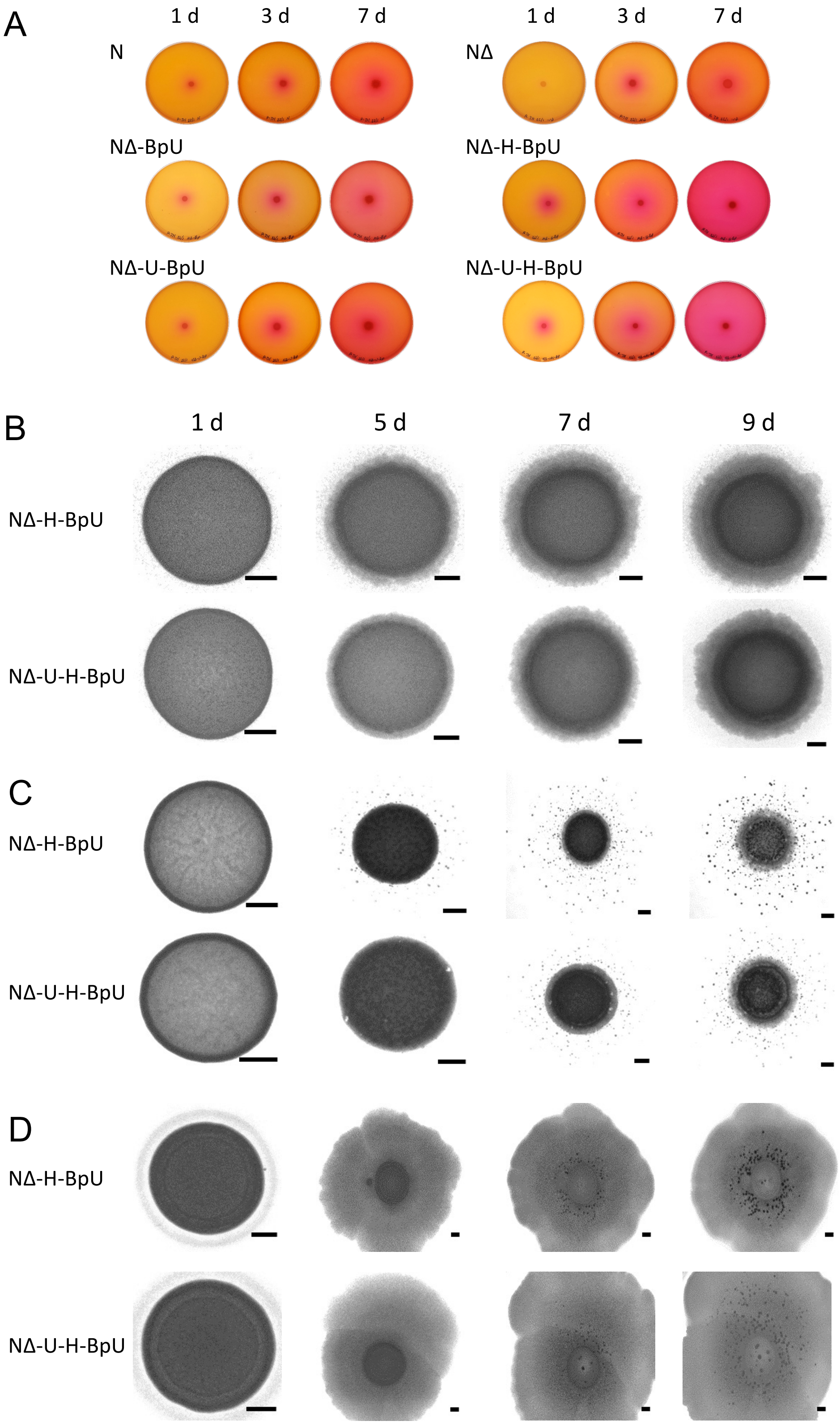

Additional file 3. Engineered urease and biomineralization activity in *B. subtilis* NCIB3610. (A) urease activity assay on LBC medium containing urea, xylose and phenol red. Cells were spotted onto the centre of the plate and photographed over seven days of incubation at 30°C as indicated above. Urease activity was observed as pink colouration of the agar. **(B-D)** Biomineralisation assays on LBC medium (B), B4 medium (C) or LBGMC biofilm-promoting medium (D), all supplemented with urea and xylose. Cells were spotted onto the centre of the agar, incubated at 30°C for up to 9 days and imaged with a stereomicroscope at the time points indicated above in panel B. The image area shown varies between days to allow visualisation of mineral crystals outside the colony area in panel C or to accommodate the increased area of growth in panel D. Scale bars represent 1 mm in size. Representative results of two independent repeats are shown. In all panels, the strain nomenclature is as follows: wild-type *B. subtilis* NCIB3610 (N); its isogentic *ureABC* deletion strain (NΔ); derived strains containing expression constructs for *B. paralicheniformis ureABCEFGD* (BpU), *ureH* (H), UT (U) or combinations thereof.
