# Supplementary material for: Genetic optimisation of bacteria-induced calcite precipitation in Bacillus subtilis
Source: Microb Cell Fact. 2021 Nov 18;20:214. doi: 10.1186/s12934-021-01704-1 (PMC8600894; doi:10.1186/s12934-021-01704-1)
Supplement: Supplementary file 4 — Additional file 4. Effects of dtlABCDE deletion on the cell surface charge of B. subtilis. (A) Zeta potential determination of B. subtilis W168 and its isogenic dlt deletion strain. (B) Cytochrome C binding by the same two strains. (C) Nisin-dependent killing of the two strains. [file 12934_2021_1704_MOESM4_ESM.pdf]

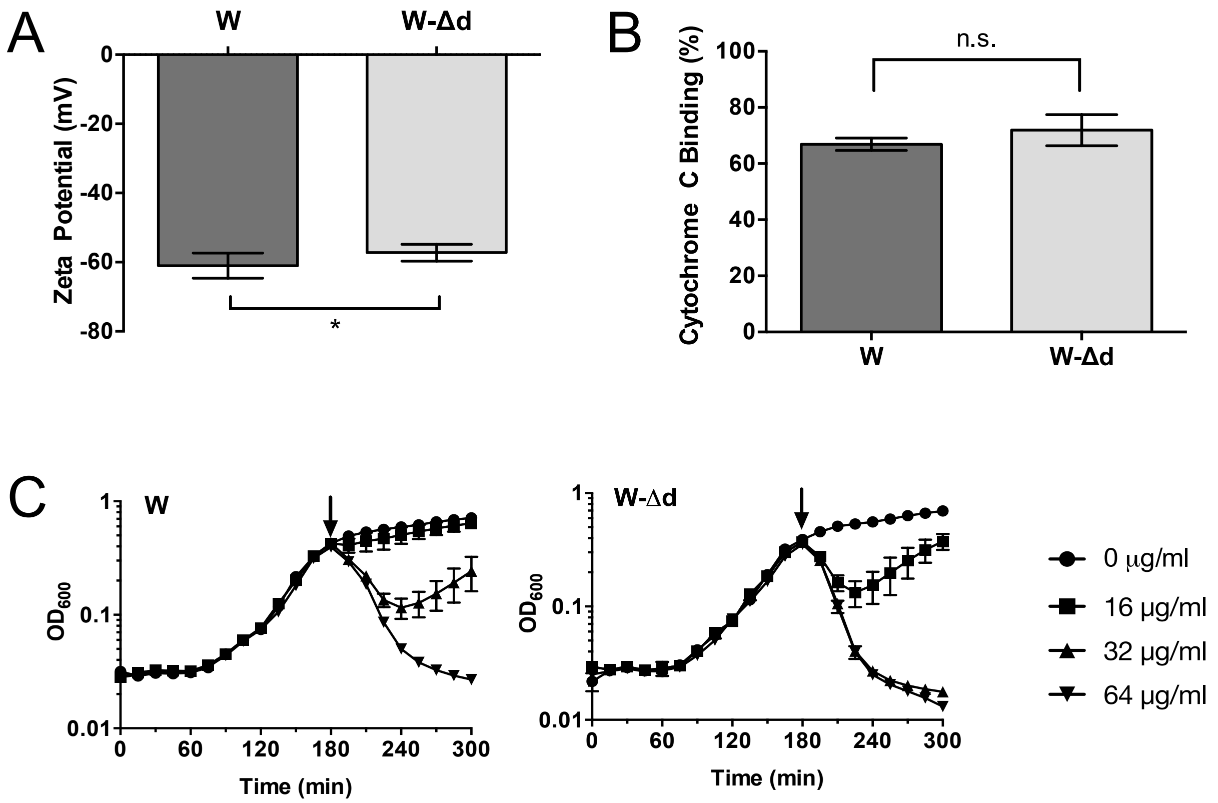

Additional file 4. Effects of *dtlABCDE* deletion on the cell surface charge of *B. subtilis*. Cells of wild-type *B. subtilis* W168 (W) or an isogenic *dltABCDE* deletion strain were subjected to surface charge analyses. **(A)** Zeta potential measurements. Cells were grown to exponential phase (OD_{600} = 0.5-1), harvested, washed and resuspended in dH_2O to OD_{600} = 0.5, and the zeta potential measured on a Zetasizer Nano ZS at 25 °C. Data are shown as mean ± standard deviation of three biological repeats, each measured in technical triplicates. n.s. shows $p>0.05$ and * shows $p\leq0.05$ from an un-paired t-test analysis. **(B)** Cytochrome C binding assay. Overnight cultures were resuspended in MOPS/NaOH [pH7] to OD_{600} = 2.5 and incubated with 250 µg/ml cytochrome C for 10 minutes at room temperature. Percentage binding was calculated as absorbance difference of the supernatant relative to samples without bacteria. Data are shown as mean ± standard deviation of three to four biological repeats; n.s. $p>0.05$ in an unpaired t-test analysis. **(C)** Nisin-dependent killing assay. Cells were grown in LB medium in a Tecan microplate reader to OD_{600} = 0.4-0.5. At the time point shown by the arrows, nisin was added at the indicated concentrations and OD_{600} monitored over time. Data are shown as mean and standard deviation of three technical repeats and are representative of three biological repeats.
