# Supplementary material for: Genetic optimisation of bacteria-induced calcite precipitation in Bacillus subtilis
Source: Microb Cell Fact. 2021 Nov 18;20:214. doi: 10.1186/s12934-021-01704-1 (PMC8600894; doi:10.1186/s12934-021-01704-1)
Supplement: Supplementary file 5 — Additional file 5. Primers used in this study. Names, use and sequence of oligonucleotides used as primers in this study. [file 12934_2021_1704_MOESM5_ESM.docx]

**Additional file 5. Primers used in this study.**

| **Name** | **Description** | **Sequence 5’ → 3’ *^a^*** |
| --- | --- | --- |
| SG0144 | kan^r^ cassette LFH fwd | CAGCGAACCATTTGAGGTGATAGG |
| SG0145 | kan^r^ cassette LFH rev | CGATACAAATTCCTCGTAGGCGCTCGG |
| SG0146 | kan^r^ cassette check fwd | CATCCGCAACTGTCCATACTCTG |
| SG0147 | kan^r^ cassette check rev | CTGCCTCCTCATCCTCTTCATCC |
| SG0148 | *lacA* integration check fwd | GCATACCGGTTGCCGTCATC |
| SG0149 | *lacA* integration check rev | GAACTACATGCACTCCACAC |
| SG0508 | SpeI + RBS + *ureABCEFGD*^Sp^ fwd | tttaa**ACTAGTaaggaggacaaac**TTGCATTTAAATCCAGCAGAGAAAG |
| SG0509 | SpeI + *ureABCEFGD*^Sp^ rev | tttaa**ACTAGT**TTAAACGATATAGTTAGCAAATTCATCC |
| SG0510 | Up-fwd *ureABC* W168 LFH | TACGGAAGAGGCAGGAAAC |
| SG0511 | Up-rev *ureABC* W168 LFH | *cctatcacctcaaatggttcgctg*CGCTGCAAAAATGAGCAATTTC |
| SG0512 | Do-fwd *ureABC* W168 LFH | *cgagcgcctacgaggaatttgtatcg*GCCAGCCTGTCGATTATGTC |
| SG0513 | Do-rev *ureABC* W168 LFH | GCAGTTGGCAAATATTCTTTCG |
| SG0526 | zeo^r^ cassette LFH fwd | *cagcgaaccatttgaggtgatagg*GGCTTTTTATATGTGTTACTCTACATACAGAAAGG |
| SG0527 | zeo^r^ cassette LFH rev | *cgatacaaattcctcgtaggcgctcgg*CAGTCGGCATTATCTCATATTATAAAAGCC |
| SG0532 | Up-fwd *ureABC* W168 outside flank check | AGGATATCGGGAAAGCCAAG |
| SG0533 | Do-rev *ureABC* W168 outside flank check | GAGTCTGTCTGCTGCAAATC |
| SG0634 | MLS^r^ cassette LFH fwd | *cagcgaaccatttgaggtgataggg*ATCCTTTAACTCTGGCAACCCTC |
| SG0635 | MLS^r^ cassette LFH rev | *cgatacaaattcctcgtaggcgctcgg*GCCGACTGCGCAAAAGACATAATCG |
| SG0646 | Biobrick prefix + RBS + UT^Bp^ fwd | tttaa***GAATTCGCGGCCGCTTCTAGAG*aaggaggacaaac**ATGCGAAAAAACAAACATCATTTATTAAG |
| SG0647 | Biobrick suffix + UT^Bp^ rev | tttaa***CTGCAGCGGCCGCTACTAGTA***TTATAAGTTTTGCAGAACCTTTCTTG |
| SG0648 | XbaI + RBS + *ureH*^Bp^ fwd | tttaa**TCTAGAaaggaggacaaac**GTGGAAGGTACATTATTCTCG |
| SG0649 | Biobrick suffix + *ureH*^Bp^ rev | tttaa***CTGCAGCGGCCGCTACTAGTA***TTAAATCCAAAGGTTAAATAAACCC |
| SG0650 | *ureH*^Bp^ QuikChange fwd | CATTGGAATCCCCTTTGTGCTGAGTAAGAAGCGAG |
| SG0651 | *ureH*^Bp^ QuikChange rev | GCACAAAGGGGATTCCAATGATTGTTGTAAAGAAAAGCATAC |
| SG0652 | BsmBI w/ BamHI overhang + RBS + *ureABCEFGD*^Bp^ fwd | tttaa**CGTCTCGGATCCaaggaggacaaac**ATGCAACTATTACCGCGTGAAG |
| SG0653 | BsmBI w/ EcoRI overhang + *ureABCEFGD*^Bp^ rev | tttaa**CGTCTCGAATTC**TTAATATTTTCTTAAAAAACTCGGGG |
| SG0891 | Up-fwd *dltABCDE* W168 LFH | CTGCTGCTGGCACAAATATAG |
| SG0892 | Up-rev *dltABCDE* W168 LFH | *cctatcacctcaaatggttcgctg*CGGATAAGTTTCCGCATGTG |
| SG0893 | Do-fwd *dltABCDE* W168 LFH | *cgagcgcctacgaggaatttgtatcg*GATTATGACAGGTTATTCGAGC |
| SG0894 | Do-rev *dltABCDE* W168 LFH | GATGACTGAATTGCGCGTAATG |
| SG0895 | Up-fwd *dltABCDE* W168 outside flank check | GCTCTGCATATTGATCTTATCTG |
| SG0896 | Do-rev *dltABCDE* W168 outside flank check | GTTACAGGTGACTATCAAACTG |
| SG1009 | Up-fwd *epsH* W168 LFH | CACTTGTGCTCGTGTCAGG |
| SG1010 | Up-rev *epsH* W168 LFH | *cctatcacctcaaatggttcgctg*CAGCGACTAACAGACTAACC |
| SG1011 | Do-fwd *epsH* W168 LFH | *cgagcgcctacgaggaatttgtatcg*GTATCAGCGGGTGATCGAG |
| SG1012 | Do-rev *epsH* W168 LFH | CATTGACTGCGGCTGTTACG |
| SG1013 | Up-fwd *tasA* W168 LFH | CTCCAATCAAATCGGCGATTC |
| SG1014 | Up-rev *tasA* W168 LFH | *cctatcacctcaaatggttcgctg*GCAGAAGCAACTCCTAAACTC |
| SG1015 | Do-fwd *tasA* W168 LFH | *cgagcgcctacgaggaatttgtatcg*CATACTGATAAAGATGGTTACGTG |
| SG1016 | Do-rev *tasA* W168 LFH | CGGAACAGGCGGTTAACAG |
| SG1021 | Up-fwd *epsH* W168 outside flank check | CTCAACTTGGCTACGATGCAC |
| SG1022 | Do-rev *epsH* W168 outside flank check | GTTCAACATTATACATCGGGAC |
| SG1023 | Up-fwd *tasA* W168 outside flank check | GAAAGGCACTGTATGCTCAC |
| SG1024 | Do-rev *tasA* W168 outside flank check | GAAAGCGGACTTAAGCGTATG |

*^a^* Restriction sites are in uppercase bold; overhangs for the BioBrick cloning standard (RFC10) are in uppercase bold and italicised; Added ribosome binding sites are in lowercase bold and underlined; long flanking homology (LFH) PCR overhangs are in lowercase underlined and italicised; the QuikChange point mutation site is double underlined.
